# Supplementary material for: A self-limiting sterile insect technique alternative for Ceratitis capitata
Source: BMC Biol. 2025 Apr 12;23:97. doi: 10.1186/s12915-025-02201-2 (PMC11993972; doi:10.1186/s12915-025-02201-2)
Supplement: Supplementary file 1 — Additional file 1: Figures S1-9; Tables S1-7. Figure S1. Integrations of piggyBac constructs. Figure S2. Optimisation of the CRISPR/Cas9 toolkit against the white eye gene. Figure S3. Fluorescence of Cas9-harbouring female ovaries. Figure S4. Characterisation of gRNA efficiency in F1 trans-heterozygotes. Figure S5. Fitness of F1 trans-heterozygotes. Figure S6. Fitness of F1 trans-heterozygotes with varied sex-conversion rates. Figure S7. Fitness of piggyBac Cas9 and dgRNA-harbouring strains. Figure S8. Protein alignment of ß-tubulin85D in Drosophila melanogaster and the selectedß-tubulin65B Ceratitis capitata showed a 73.90% similarity. Figure S9. Characterisation of trans-heterozygous F1 males. Table S1. Genomic integration annotations of piggyBac constructs. Table S2. Eye colour phenotypes of DsRed-/GFP+ F1 progeny. Table S3. Sex phenotypes of DsRed-/GFP+ F1 progeny . Table S4. Morphology of F1 intersex internal genitalia. Table S5. Crosses performed in line egg-adult assay. Table S6. Primer summary. Table S7. gRNA summary. [file 12915_2025_2201_MOESM1_ESM.pdf]

A self-limiting Sterile Insect Technique alternative for *Ceratitis capitata*

Supplementary Materials

Serafima Davydova[1], Junru Liu[2], Yiran Liu[3], Kavya Prince[1], Jonathan Mann[1], Nikolay P. Kandul[2], W. Evan Braswell[4], Jackson Champer[3], Omar S. Akbari[2], Angela Meccariello[1]*

1.Department of Life Sciences, Imperial College London, London, SW7 2AZ, United Kingdom

2.School of Biological Sciences, Department of Cell and Developmental Biology, University of California, San Diego, La Jolla, CA 92093, United States of America

3.Center for Bioinformatics, School of Life Sciences, Center for Life Sciences, Peking University, Beijing, 100871, China

4.USDA APHIS PPQ Science and Technology Insect Management and Molecular Diagnostic Laboratory, 22675 North Moorefield Road, Edinburg, Texas, 78541, United States of America

* Corresponding author

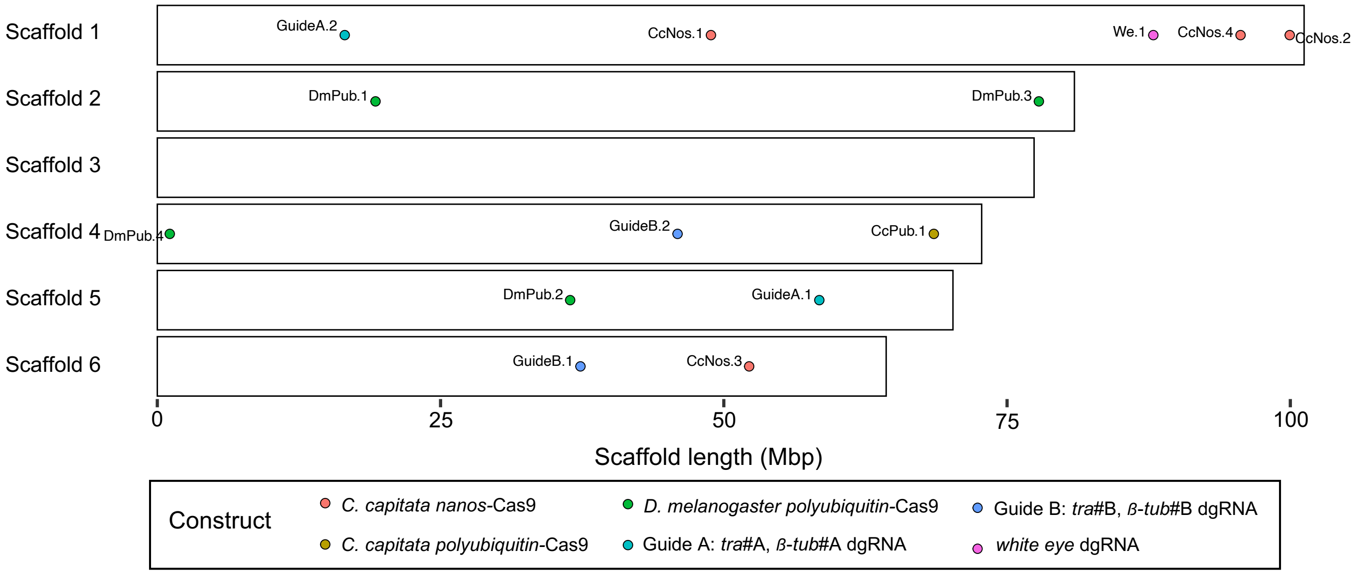

Figure S1. Integrations of *piggyBac* constructs
The map depicting the genomic integrations for the 3 Cas9 and 3 double-guide RNA (dgRNA) *piggyBac* constructs used in this study. As a result of germline transformation, 14 strains with unique integrations were established and characterised via inverse PCR using the GenBank GCA_905071925.1 *C. capitata* genome re-assembly. Constructed in RStudio.

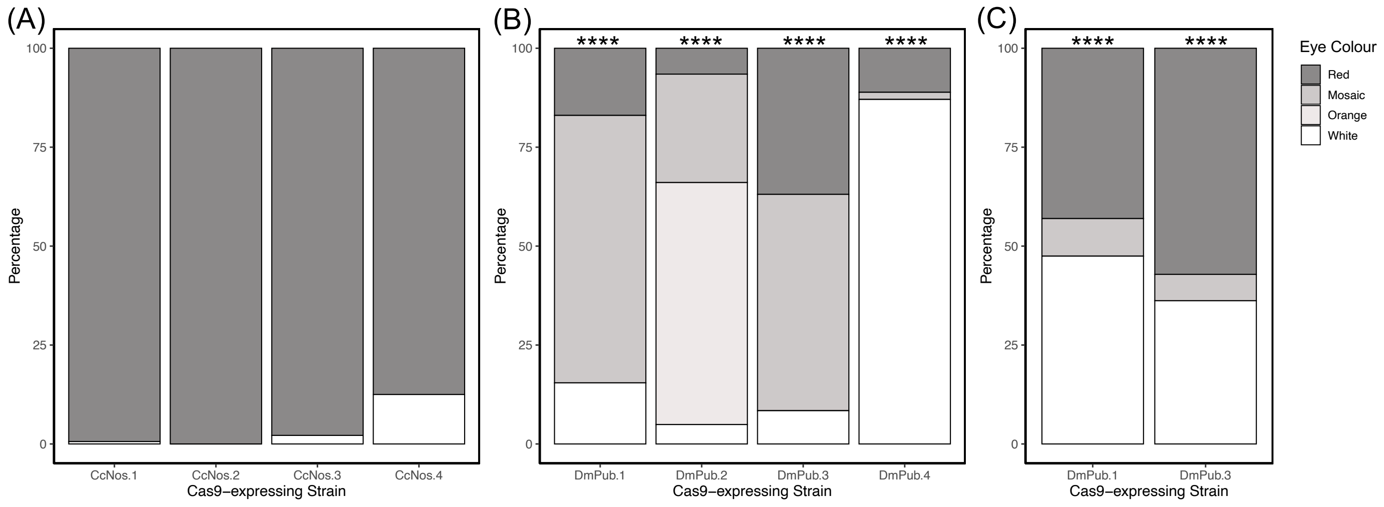

Figure S2. Optimisation of the CRISPR/Cas9 toolkit against the *white eye* gene

Stack graphs showing eye colour percentages of (A) *Ceratitis capitata nanos*-Cas9 F2, (B) *Drosophila melanogaster polyubiquitin*-Cas9 F1, and (C) *D. melanogaster polyubiquitin*-Cas9 F2 progeny. Individuals were exclusively screened for eye colour and scored as 'red', 'mosaic', 'orange' or 'white'. (B) F1 population is the direct result of crosses between Cas9-harbouring females with dgRNA-harbouring (We.1) males. (A, C) F2 populations are a result of crosses between DsRed+/GFP+ red-eyed F1 females and males from the homozygous *white eye* mutant (-/-) strain. Significance levels for the chi-squared test are indicated as follows: $p < 0.05$ = *, $p < 0.01$ = **, $p < 0.001$ = *** and $p < 0.0001$ = ****. Constructed in RStudio.

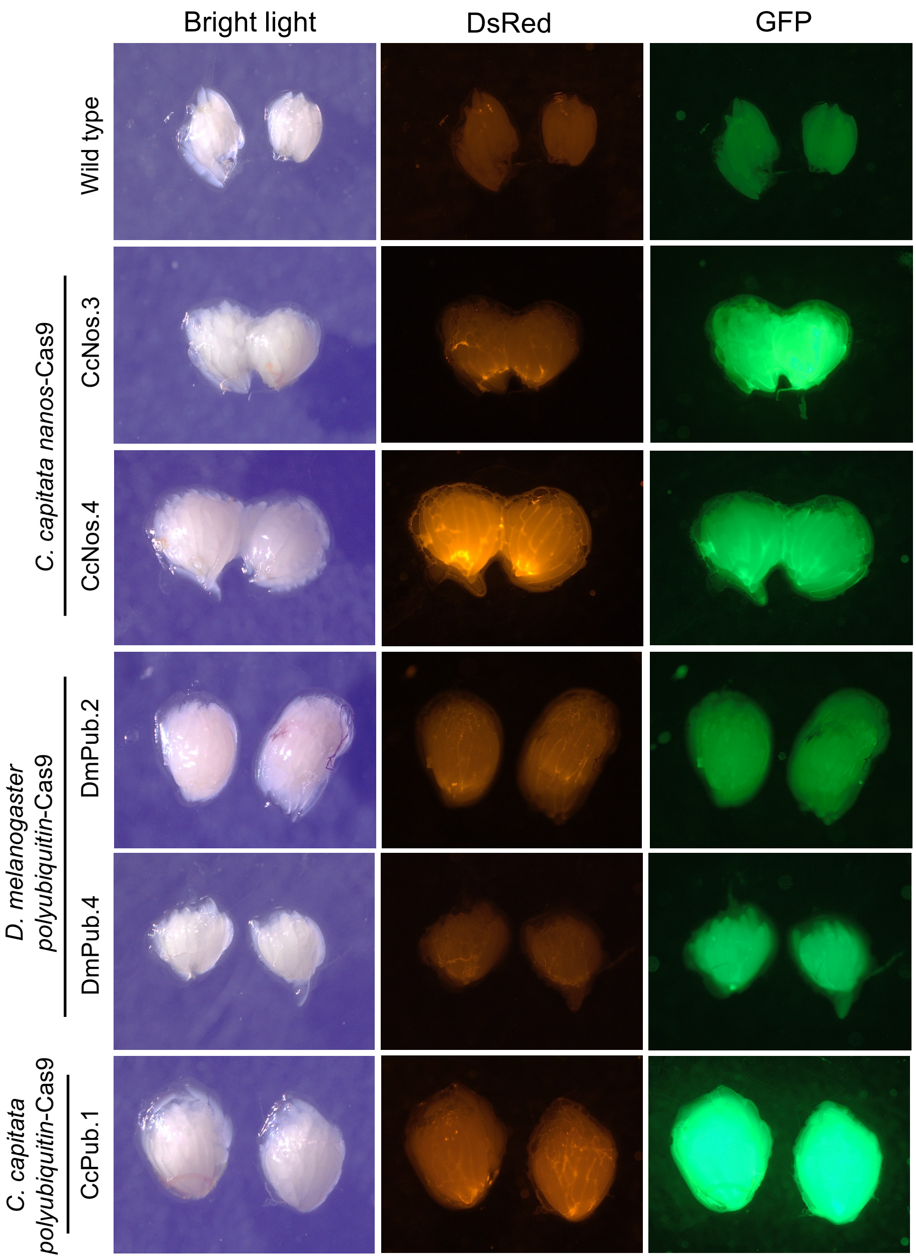

Figure S3. Fluorescence of Cas9-harbouring female ovaries
Representative ovaries, dissected from sexually mature females, were imaged under red fluorescence protein (RFP) and green fluorescence protein (GFP) filters, in addition to bright light with standardised settings. All Cas9 cassettes possess a constitutively-expressed DsRed transformation marker and are equipped with GFP, which is linked to the upstream Cas9 via a T2A peptide. Dissections were performed on homozygous females from CcNos.3, CcNos.4, DmPub.2 and CcPub.1 strains, whilst for DmPub.4 heterozygous females were dissected instead due to a lack of homozygous individuals in the strain.

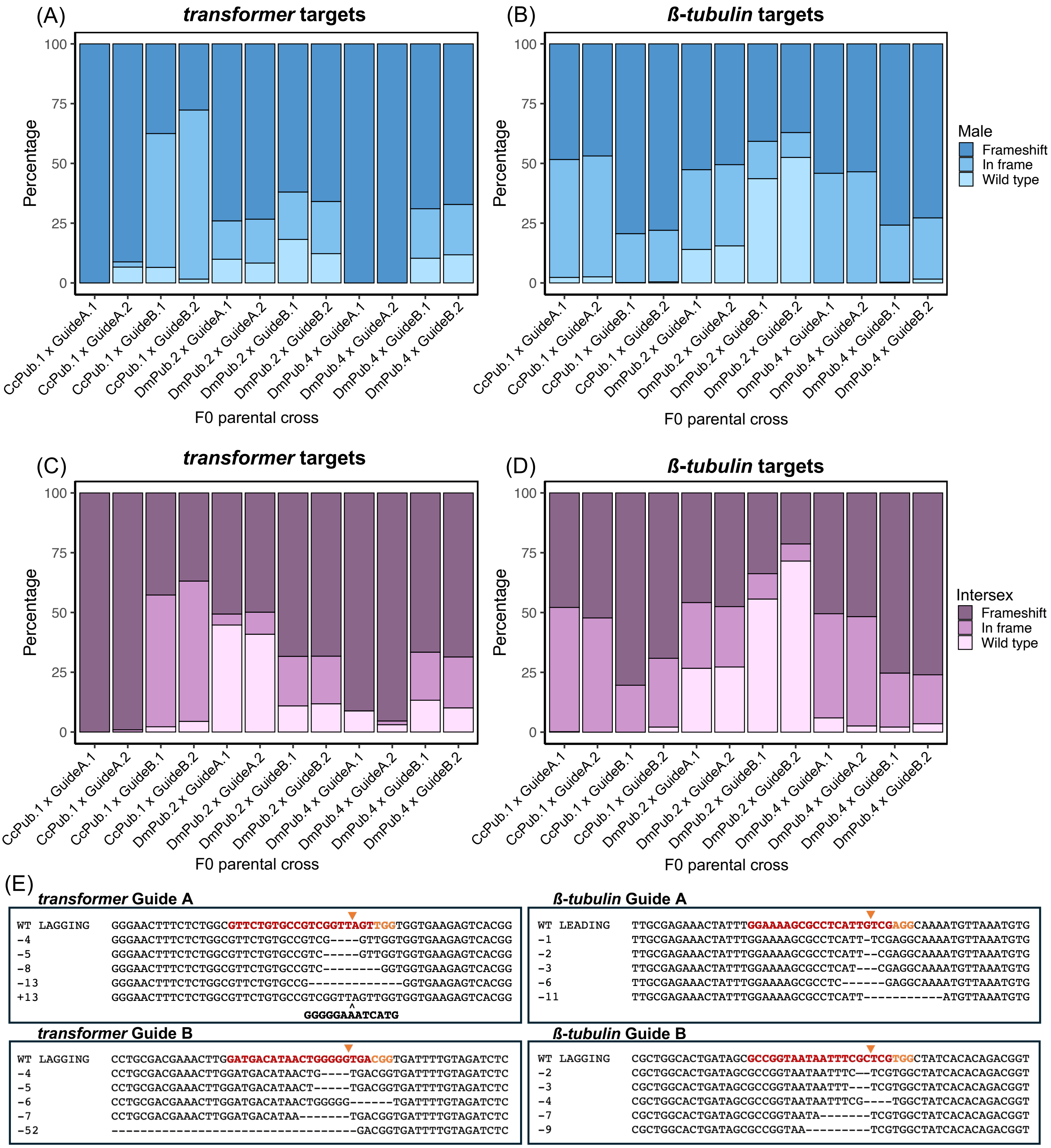

Figure S4. Characterisation of gRNA efficiency in F1 trans-heterozygotes
(A-D) Stack graphs showing mean predicted sequence identities for (A, C) *transformer* and (B, D) *ß-tubulin* gRNA targets in DsRed+/GFP+ F1 (A, B) males and (C, D) intersexes. (A-D) The averages were obtained from 3-11 sequenced males or intersexes per F0 cross. DECODR v3.0 (65) was used for sequence deconvolution whereby individual sequence identities were broken down into wild-type, in-frame indel or frameshift by percentage. (E) Example indels identified through sequence deconvolution for the 4 used gRNAs, whereby sequences are shown with 5'-3' gRNA direction.

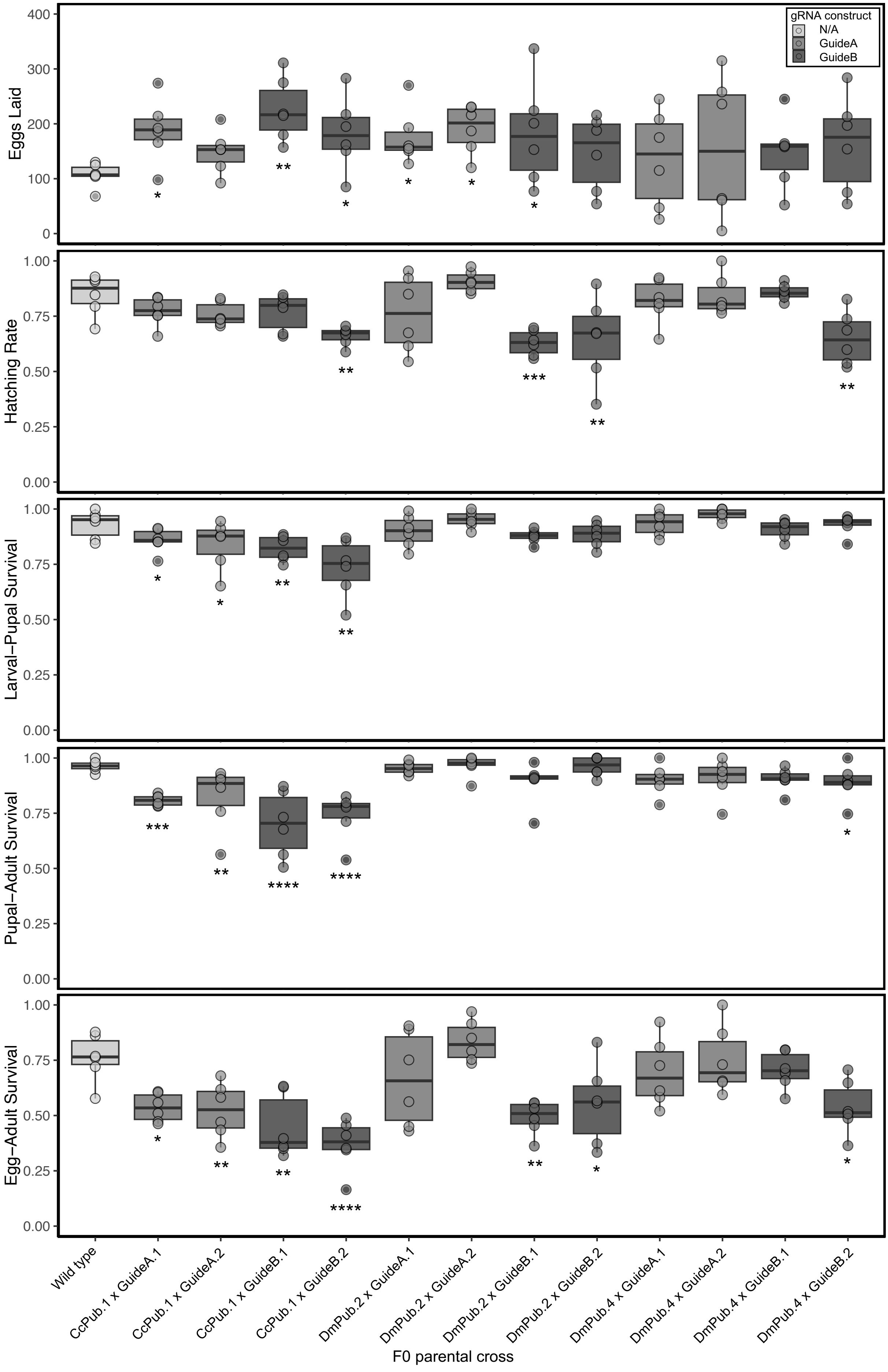

Figure S5. Fitness of F1 trans-heterozygotes

Boxplots showing egg-adult survival of F1 trans-heterozygotes compared to wild-type controls measured via eggs laid with corresponding hatching rates, hatched larval-pupal, pupal-adult and total egg-adult recovery rates. Total eggs assessed were collected within a 5-hour period from 6 replicate crosses between 5 dgRNA-harbouring males and 15 Cas9-harbouring females. The boxes represent the interquartile ranges, the lines represent the mean values, the whiskers represent minima and maxima, and the dots represent raw replicate values with outliers of 1.5 times interquartile range found outside the whiskers. The statistically significant wild-type – transgenic differences are displayed on the bar charts as follows: $p < 0.05$ = *, $p < 0.01$ = **, $p < 0.001$ = *** and $p < 0.0001$ = **** (Dunn's test). Constructed in RStudio.

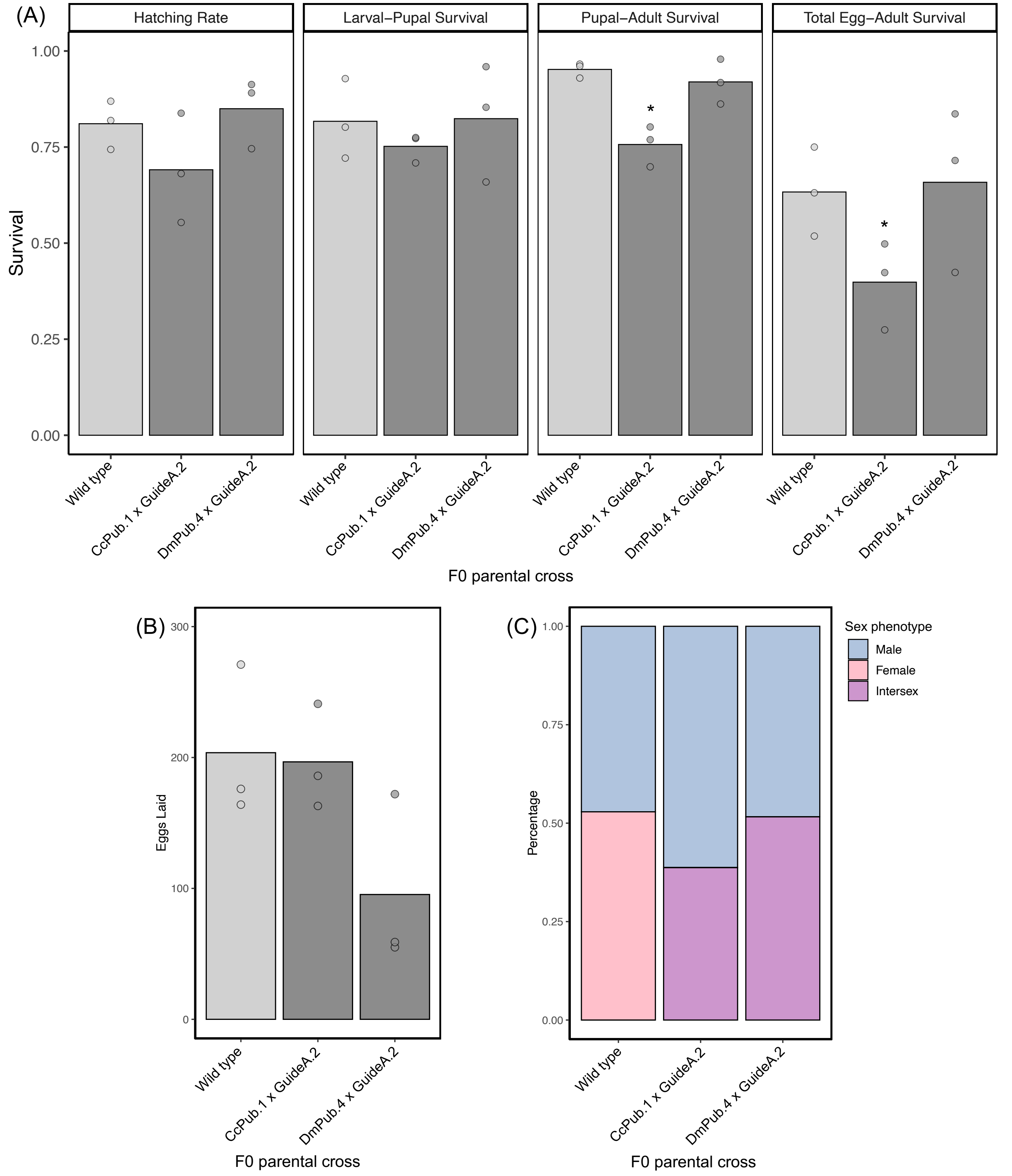

Figure S6. Fitness of F1 trans-heterozygotes with varied sex-conversion rates

(A) Bar charts showing egg-adult survival of F1 trans-heterozygotes compared to wild-type controls showing hatching rates, hatched larval-pupal, pupal-adult and total egg-adult recovery rates. (B) A bar chart showing corresponding eggs laid, collected within a 5-hour period from triplicates crosses between 10 dgRNA-harbouring males and 20 Cas9-harbouring females. (C) A stack graph showing the corresponding mean adult phenotypes which were scored as 'male', 'intersex' or 'female'. Flies were screened for DsRed and GFP and all non-wild-type F1 progeny was DsRed+/GFP+. (A-B) The bar levels represent the mean values, and the dots represent raw replicate values. The statistically significant wild-type – transgenic differences are displayed on the bar charts as follows: $p < 0.05$ = * (Dunn's test). Constructed in RStudio.

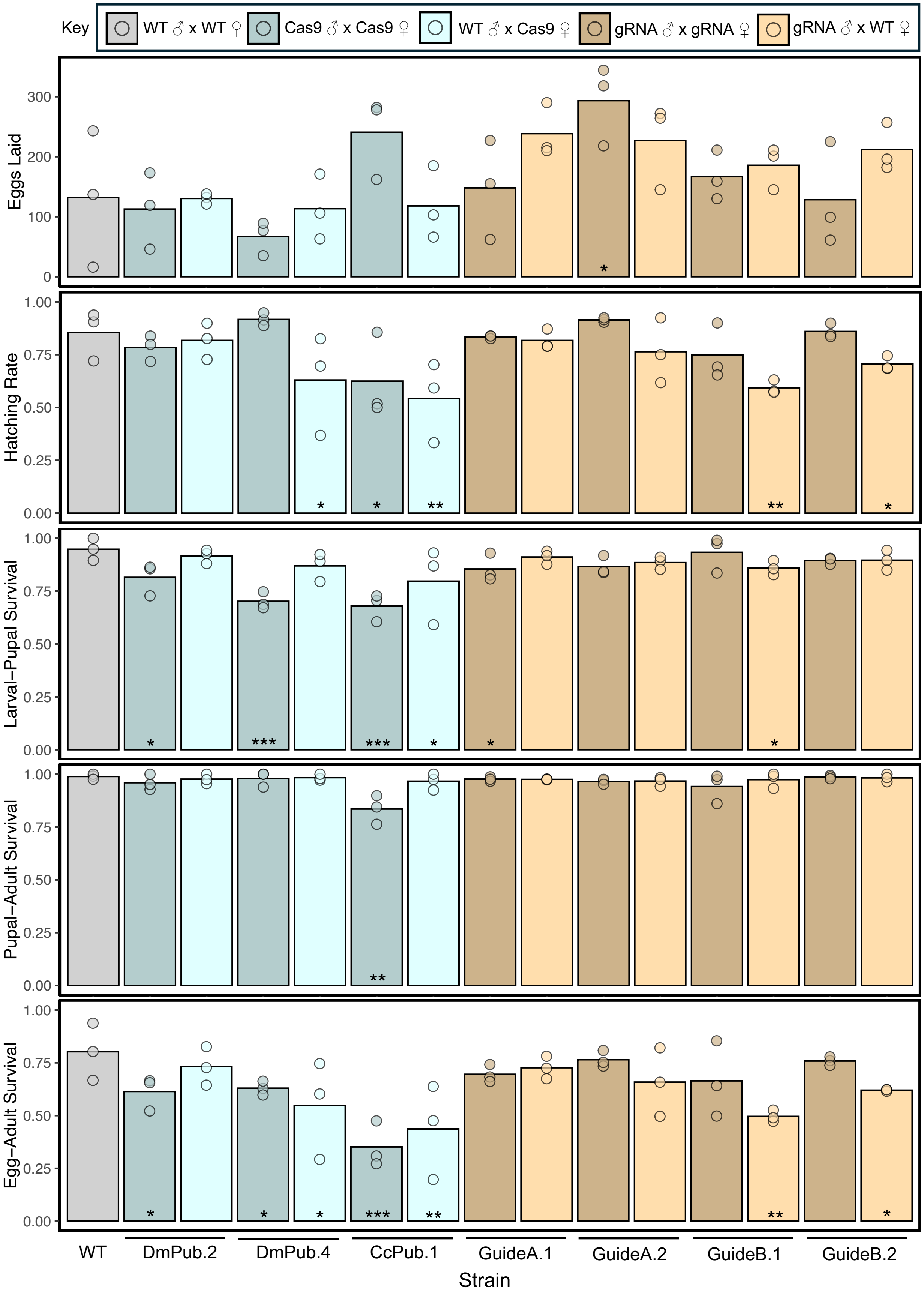

Figure S7. Fitness of *piggyBac* Cas9 and dgRNA-harbouring strains

Bar charts showing transgenic strain egg-adult survival compared to wild-type controls measured via eggs laid with corresponding hatching rates, hatched larval-pupal, pupal-adult and total egg-adult recovery rates. Total eggs assessed were collected within a 5-hour period from triplicate crosses between 10 males and 20 females. Each Cas9-harbouring strain (DmPub.2, DmPub.4, CcPub.1), depicted in blue, was subjected to sibling crosses and crosses of transgenic females with wild-type males. Each dgRNA-harbouring strain (GuideA.1-2; GuideB.1-2), depicted in beige, was subjected to sibling crosses and crosses of transgenic males with wild-type females. The bar levels represent the mean values, and the dots represent raw replicate values. The crosses performed are additionally summarised in Supplementary Table 5. The statistically significant wild-type – transgenic differences are displayed on the bar charts as follows: $p < 0.05$ = *, $p < 0.01$ = ** and $p < 0.001$ = *** (Dunn's test). Constructed in RStudio.

```
▶ Dm_CG9359_βTub85D                 1   MREIVHIQAGQCGNQIGGKFWEVISDEHCID-ATGTYYGDSDLQLERINVYYNEATGAKY  59
                                        MREI+ IQ G  GN IG  FW VIS EH +D ++G + G S LQLERINV++N     ++
▶ Medfly_ LOC10...87_betaTub65B     1   MREIITIQIGASGNSIGDAFWHVISHEHGVDYSSGRFQGTSALQLERINVFFNATASKRF  60

▶ Dm_CG9359_βTub85D                 60  VPRAILVDLEPGTMDSVRSGAFGQIFRPDNFVFGQSGAGNNWAKGHYTEGAELVDSVLDV  119
                                          RAI +D E  T++++    +    ++RP+NFV G   AGNN+A+G++T+GAE++D+VL+
▶ Medfly_ LOC10...87_betaTub65B     61  YARAICIDTEATTIENLHKRS--SLYRPENFVAGTDSAGNNFARGYHTDGAEILDAVLNA  118

▶ Dm_CG9359_βTub85D                 120 VRKESEGCDCLQGFQLTHSLGGGTGSGMGTLLISKIREEYPDRIMNTFSVVPSPKVSDTV  179
                                        +R+E+E   D LQGFQ+ HS+GGGTGSG+  L+++ + +EYPD +++ +   +PSP++S  V
▶ Medfly_ LOC10...87_betaTub65B     119 IRREAESVDSLQGFQILHSIGGGTGSGLTALVMNALIDEYPDNLISNYVSIPSPQMSQVV  178

▶ Dm_CG9359_βTub85D                 180 VEPYNATLSVHQLVENTDETYCIDNEALYDICFRTLKLTTPTYGDLNHLVSATMSGVTTC  239
                                        VEPYNA LS+   L+ N+   T+C+DNEAL+ I   R LK+     Y   +NH+V+ TMSG+TTC
▶ Medfly_ LOC10...87_betaTub65B     179 VEPYNALLSMPSLIHNSHLTFCLDNEALFQIVSRNLKMAVSGYEHINHIVALTMSGITTC  238

▶ Dm_CG9359_βTub85D                 240 LRFPGQLNADLRKLAVNMVPFPRLHFFMPGFAPLTSRGSQQYRALTVPELTQQMFDAKNM  299
                                        LRFPGQLNA LRKL VNM+PFP+LHF +PGFAPL S    +++    TV EL QQ+F + N+
▶ Medfly_ LOC10...87_betaTub65B     239 LRFPGQLNAGLRKLYVNMIPFPQLHFLIPGFAPLISCKQKEFSQGTVGELVQQIFSSNNL  298

▶ Dm_CG9359_βTub85D                 300 MAACDPRHGRYLTVAAIFRGRMSMKEVDEQMLNIQNKNSSFFVEWIPNNCKTAVCDIPPR  359
                                        + A D RHG+ LT A IFRGRMS +EVD+ M N++NKN S FVEWIPNN KTA+CDIPPR
▶ Medfly_ LOC10...87_betaTub65B     299 LCAVDLRHGKLLTAAGIFRGRMSPREVDQLMTNVRNKNLSSFVEWIPNNIKTAICDIPPR  358

▶ Dm_CG9359_βTub85D                 360 GLKMSATFIGNSTAIQELFKRVSEQFTAMFRRKAFLHWYTGEGMDEMEFTEAESNMNDLV  419
                                        GLKMSATFIGN++AI +L +R+ +   + M RRKA LHWYTGEGM+E EF +A+ ++ D +
▶ Medfly_ LOC10...87_betaTub65B     359 GLKMSATFIGNTSAIHKLLQRILDGSSIMLRRKAHLHWYTGEGMEEQEFIDAQKDLQDTL  418

▶ Dm_CG9359_βTub85D                 420 SEYQQYQEATADE  432
                                          Y++  E  +++
▶ Medfly_ LOC10...87_betaTub65B     419 DLYKRSGEDDSEK  431
```

Figure S8. Protein alignment of *ß-tubulin85D* in *Drosophila melanogaster* and the selected *ß-tubulin65B Ceratitis capitata* showed a 73.90% similarity

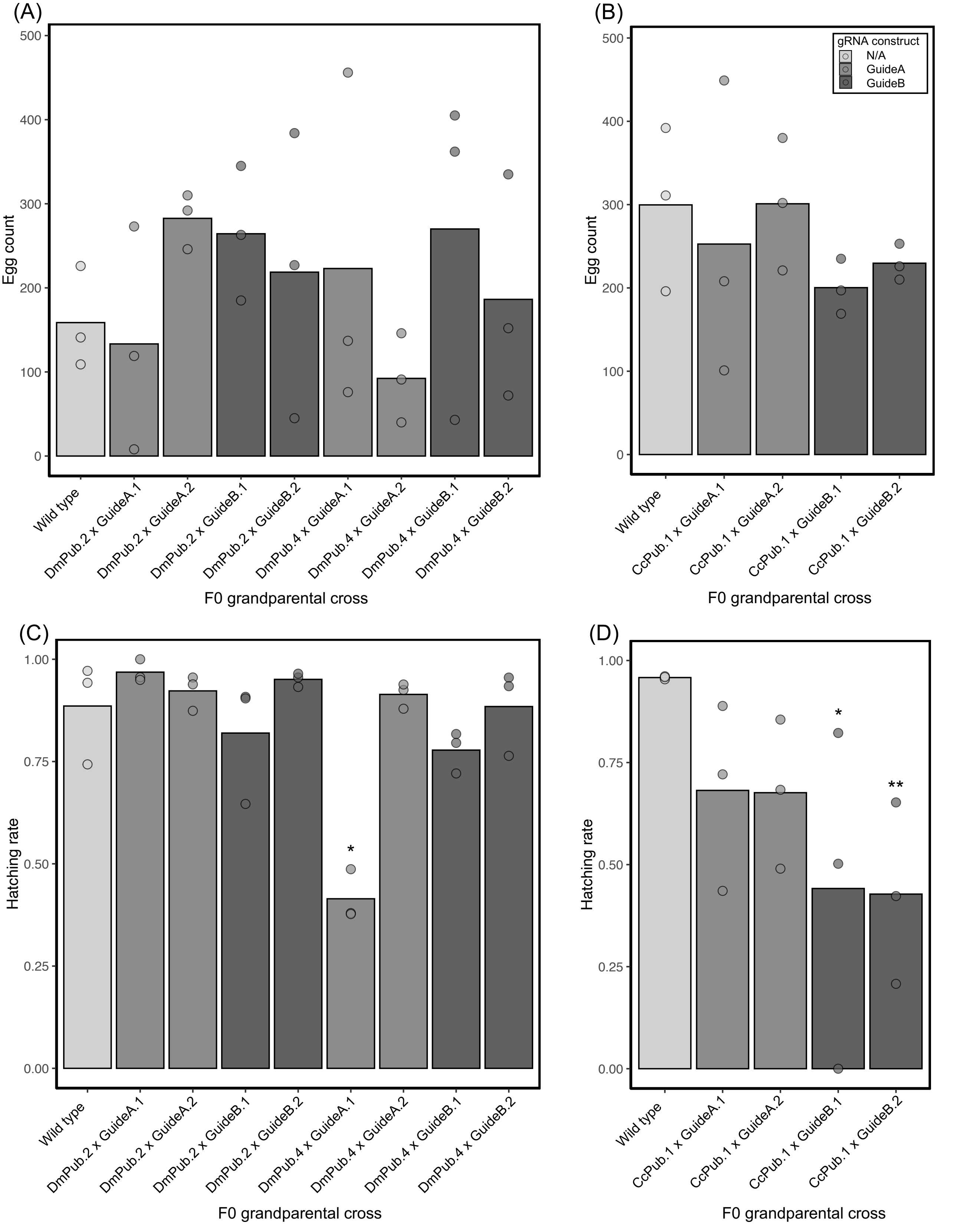

Figure S9. Characterisation of trans-heterozygous F1 males

Bar charts showing egg laying (A, B) and corresponding egg hatching (C, D) rate comparisons between wild-type sibling crosses with DsRed+/GFP+ F1 males crossed with wild-type females. Egg laying and egg hatching were measured separately for the wild-type-crossed F1 males from (A, C) F0 *Drosophila melanogaster polyubiquitin*-Cas9 (DmPub.1-4)-harbouring females with the dgRNA (GuideA.1-2; GuideB.1-2)-harbouring males; and (B, D) F0 *Ceratitis capitata polyubiquitin*-Cas9 (CcPub.1)-harbouring females with the dgRNA (GuideA.1-2; GuideB.1-2)-harbouring males. (A-D) Total eggs assessed were collected within a 5-hour period from triplicate crosses between 10 males and 20 females. The bar levels represent the mean values, and the dots represent raw replicate values. The statistically significant wild-type – transgenic differences are displayed on the bar charts as follows: $p < 0.05$ = * and $p < 0.01$ = ** (Dunn's test). Constructed in RStudio.

Table S1. Genomic integration annotations of *piggyBac* constructs

The genomic sequences flanking the *piggyBac* constructs as determined through inverse PCR and visually represented in Supplementary Figure 1. Altogether 14 strains with unique integrations were established in this study.

Transformation Construct	Strain Name	Scaffold	Annotation	Integration Site
white eye dgRNA	We.1	1	87 907 601	CCAGCAGTGATTAA-PB-TTAACGTAGGTACG
C. capitata nanos-Cas9	CcNos.1	1	48 864 350	TCGAGCATTGTTAA-PB-TTAAGATTGTGTGC
	CcNos.2	1	99 947 453	GAGGTGGATATTAA-PB-TTAATATTATTTCC
	CcNos.3	6	52 237 118	AAAACACCCTTTAA-PB-TTAAAACTTCTTTA
	Cc.Nos.3	1	95 608 274	TGAAAGCTACTTAA-PB-TTAACTTCGCATTT
D. melanogaster polyubiquitin-Cas9	DmPub.1	2	19 259 842	TGTGGCGTGTTTAA-PB-TTAACTAACTACGT
	DmPub.2	5	36 439 356	GAAGTTGAATTTAA-PB-TTAAAGCGACAAAC
	DmPub.3	2	77 806 718	CACATCTCTATTAA-PB-TTAACAGCTATAAT
	DmPub.4	4	1 101 559	ACTTAACGAATTAA-PB-TTAATGGCAACGAT
C. capitata polyubiquitin-Cas9	CcPub.1	4	68 536 031	CAACATATTTTTAA-PB-TTAAATAAGCCCTA
GuideA: *tra*#A, *ß-tub*#A dgRNA	GuideA.1	5	58 430 586	ATCGTAAGACTTAA-PB-TTAAATCGTAAGAC
	GuideA.2	1	16 540 004	GAATACGTGTTTAA-PB-TTAAGTCTTATTTT
GuideB: *tra*#B, *ß-tub*#B dgRNA	GuideB.1	6	37 346 393	TAATCCTGCTTTAA-PB-TTAACAAGACTGGT
	GuideB.2	4	45 917 139	CGCAAATGTGTTAA-PB-TTAATTTTAAGACA

Table S2. Eye colour phenotypes of DsRed-/GFP+ F1 progeny

The Cas9-harbouring females were crossed with males from the *white eye*-dgRNA-harbouring We.1 strain. Their progeny was screened for fluorescence and classified by eye colour thereafter.

	Eye colour			
**Cross**	**Red**	**Mosaic**	**Orange**	**White**
DmPub.2 x We.1	7			
DmPub.4 x We.1		4		147
CcPub.1 x We.1				106

Table S3. Sex phenotypes of DsRed-/GFP+ F1 progeny
The Cas9-harbouring and dgRNA-harbouring strains were reciprocally crossed together, and their progeny was scored as 'Male', 'Intersex' or 'Female' post fluorescence screening.

		Sex phenotype		
**Source of Cas9**	**Cross**	**Female**	**Intersex**	**Male**
Maternal	DmPub.2 x GuideA.1	1		1
	DmPub.2 x GuideA.2			1
	DmPub.2 x GuideB.1			
	DmPub.2 x GuideB.2			
	DmPub.4 x GuideA.1		47	159
	DmPub.4 x GuideA.2		76	285
	DmPub.4 x GuideB.1		63	96
	DmPub.4 x GuideB.2		135	144
	CcPub.1 x GuideA.1		1	7
	CcPub.1 x GuideA.2		5	28
	CcPub.1 x GuideB.1		51	60
	CcPub.1 x GuideB.2		73	82
Paternal	DmPub.2 x GuideA.1			
	DmPub.2 x GuideA.2			
	DmPub.2 x GuideB.1			
	DmPub.2 x GuideB.2			
	DmPub.4 x GuideA.1	58		51
	DmPub.4 x GuideA.2	80		92
	DmPub.4 x GuideB.1	45		43
	DmPub.4 x GuideB.2	97		112
	CcPub.1 x GuideA.1	24		24
	CcPub.1 x GuideA.2	20		17
	CcPub.1 x GuideB.1	22		28
	CcPub.1 x GuideB.2	9		12

Table S4. Morphology of F1 intersex internal genitalia

Trans-heterozygous intersexes were abdominally dissected upon sexual maturation.

Parental crosses	2 full ovaries	2 malformed ovaries	1 full ovary	1 malformed ovary	Nothing	1 malformed testis	1 full testis	2 malformed testes	2 full testes	Total
GuideA x DmPub.2	2	2							1	**5**
GuideA x DmPub.4				1	2	1		1		**5**
GuideA x CcPub.1					4	1				**5**
GuideB x DmPub.2	5									**5**
GuideB x DmPub.4		1			4					**5**
GuideB x CcPub.1			3		2					**5**
**Total**	**7**	**3**	**3**	**1**	**12**	**2**	**0**	**1**	**1**	**30**

Table S5. Crosses performed in line egg-adult assay

The parental crosses for line egg-adult assay were performed in triplicates for 3 Cas9-harbouring and 4 dgRNA-harbouring strains alongside wild type controls. Both sibling and transgenic-wild type crosses were performed to mimic 2 and 1 transgene copy inheritance, respectively.

Construct type	Females	Males
Cas9	DmPub.2 homozygotes	DmPub.2 homozygotes
	DmPub.2 homozygotes	Wild type
	DmPub.4 heterozygotes	DmPub.4 heterozygotes
	DmPub.4 heterozygotes	Wild type
	CcPub.1 homozygotes	CcPub.1 homozygotes
	CcPub.1 homozygotes	Wild type
dgRNA	GuideA.1 homozygotes	GuideA.1 homozygotes
	Wild type	GuideA.1 homozygotes
	GuideA.2 homozygotes	GuideA.2 homozygotes
	Wild type	GuideA.2 homozygotes
	GuideB.1 homozygotes	GuideB.1 homozygotes
	Wild type	GuideB.1 homozygotes
	GuideB.2 homozygotes	GuideB.2 homozygotes
	Wild type	GuideB.2 homozygotes
N/A	Wild type	Wild type

Table S6. Primer summary
The primers used for F1 trans-heterozygote characterisation and plasmid cloning. The karyotyping PCR was performed with previously described primers (66), whilst all other primers were designed herein.

Purpose	Primer Name	Primer Sequence (5'-3')
Karyotyping PCR	CcYF	GCTCGAAGACATGCATTGAA
	CcYR	GACGGTAAGTGCCATTCGTT
tra target PCR	tra_F	ACAGCGGTAACAATATTACAACACGA
	tra_R	ATGTAACGATCCACTGAAGACGAG
ß-tub target PCR	btub_F	GTGTAACTGTGGAAACGGTATCATG
	btub_R	ATTCATCTGGACGCTTTCAAGGC
Cloning	1139C.C1F	TTCTCGACGGTCACGGCGGGCATGTCGACTCGAGGTTGCGAATAGTAACGCTGAAAAGGT
	1139C.C2R	CCGTCGTGGTCCTTATAGTCCATTTTGTTTAAACAATTATTACTACAGCTATCAGTATTACAATCCG
	1139A1.c1F	GATAGCGATTCGAGTTAACGGCCGGGGCGCGCCGCGTAAAACACAATCAAGTATGAGTCA
	1139D.c1R	AGGCGCTCGGTGGAGGCCTCCCAGCCCATGGTCTTCTTCTGCATTACGGGGCCG
	1139E.C1F	CGGTCACGGCGGGCATGTCGACGCGGCCGCTTAGTTCATTGCGTATAGTATATGTACACA
	1139E.C2R	CGTCGTGGTCCTTATAGTCCATATTTAAATCGAAAAGTGCTAAAATAAACGAAACATATT
	1142B-2.C1F	CACATAATTTGGGTGGCGAGCGTAAGCGACGTTTTAGAGCTAGAAATAGCAAGTT
	1142B-2.C2R	ACGGTGTACCCAGAAAGGCAACAAATTATGTGAAAAACACATAGTATTTATACCAG
	1142B-2.C3F	TGGTATAAATACTATGTGTTTTTCACATAATTTGGGTGGCGAGCGTAAGCGACGTTTTAG
	1142B-2.C4R	CTTATTTTAACTTGCTATTTCTAGCTCTAAAACGGTGTACCCAGAAAGGCAACAAATTAT
	1141C.C1F	CACATAATTTGTTCTGTGCCGTCGGTTAGTGTTTTAGAGCTAGAAATAGCAAGTT
	1141C.C2R	ACCGACAATGAGGCGCTTTTCCAAATTATGTGAAAAACACATAGTATTTATACCAG
	1141C.C3F	TGGTATAAATACTATGTGTTTTTCACATAATTTGTTCTGTGCCGTCGGTTAGTGTTTTAG
	1141C.C4R	CTTATTTTAACTTGCTATTTCTAGCTCTAAAACCGACAATGAGGCGCTTTTCCAAATTAT
	1141E.C1F	CACATAATTTGATGACATAACTGGGGGTGAGTTTTAGAGCTAGAAATAGCAAGTT
	1141E.C2R	ACCGAGCGAAATTATTACCGGCAAATTATGTGAAAAACACATAGTATTTATACCAG
	1141E.C3F	TGGTATAAATACTATGTGTTTTTCACATAATTTGATGACATAACTGGGGGTGAGTTTTAG
	1141E.C4R	CTTATTTTAACTTGCTATTTCTAGCTCTAAAACCGAGCGAAATTATTACCGGCAAATTAT
Cc nanos gDNA PCR for promoter amplification	1139C.S1F	CCCGGTCTTCTACTTGGTTGCGAATAGTAACGC
	1139C.S2R	CCTATTTCCGGTATGCATTTAATATCAAGAGATCC
	1139C.S3F	CTTACCATTGTTAATAAATTGCCAGTAACTTTTGCTT
	1139C.S4R	CCGTTGTTTTCCACGAAAATCTCTACTGTGATTTCC
White eye gDNA PCR for target selection	1142.S1F	GTCATATACCCGCGCCGCGCAAACATCTCTTG
	1142.S2R	GGGTTGTAATTGGTTGGACAAGTAGCGCCAATGC
	1142.S3F	GTCTCTGGCGTCGCTTACCCCGGTGAATTAC
	1142.S4R	CCGCTTCACCGGGTGTACCCAGAAAGGCAAC
pU6 gDNA PCR for promoter amplification	1140C.S1F	CGAAACAGCATTTTCCGAATTTGCTGCAGTG
	1140C.S2R	GGCACAAATGCACACACACATACATATATGTG
tra gDNA PCR for target selection	1135.S1F	CACTCGATATAAAAAACTCTCTTATAGGGTAATTCATTTCTCG
	1135.S2R	CAACCTCCTCTTTACTGTTGTTACTTCGATCTTTAG
	1135.S3F	AGCCTTTGTATGTATATGATACTAATCCATACATCTATGGTATCT
	1135.S4R	CCAGCATTTATCCTTGCAATCAACAGCAAATAAGAG
ß-tub gDNA PCR for target selection	1138.S1F	CAATATGCGTGAAATTATAACGATCCAAATAGGTGCGAGTGG
	1138.S2R	CAATCCAATTTATTACTTCTGATCGTTGCCTTCATTACAACCTGG

Table S7. gRNA summary
The gRNAs designed using the CHOPCHOP tool (60) (*MM, mismatches*).

gRNA	Target sequence	Genomic location	Strand	GC content (%)	Self-complementarity	MM0	MM1	MM2	MM3	Efficiency
we#1	GGGTGGCGAGCGTAAGCGAC**TGG**	seq:10579	+	70	0	1	0	0	0	49.19
we#2	GTTGCCTTTCTGGGTACACC**CGG**	seq:10808	+	55	1	1	0	0	0	59.66
ß-tub#A	GGAAAAGCGCCTCATTGTCG**AGG**	seq:744	-	55	1	1	0	0	0	59.07
ß-tub#B	GCCGGTAATAATTTCGCTCG**TGG**	seq:430	+	50	0	1	0	0	0	56.49
tra#A	GTTCTGTGCCGTCGGTTAGT**TGG**	seq:1876	-	55	0	1	0	0	1	47.6
tra#B	GATGACATAACTGGGGGTGA**CGG**	seq:1984	-	50	0	1	0	0	0	65.82
